# Cytogenetically visible copy number variations (CG-CNVs) in banding and molecular cytogenetics of human; about heteromorphisms and euchromatic variants

**DOI:** 10.1186/s13039-016-0216-1

**Published:** 2016-01-22

**Authors:** Thomas Liehr

**Affiliations:** Jena University Hospital, Friedrich Schiller University, Institute of Human Genetics, Kollegiengasse 10, D-07743 Jena, Germany

**Keywords:** Heteromorphism, Copy number variations (CNVs), Banding cytogenetics, Molecular cytogenetics, Euchromatic variants (EVs)

## Abstract

**Background:**

Copy number variations (CNVs) having no (obvious) clinical effects were rediscovered as major part of human genome in 2004. However, for every cytogeneticist microscopically visible harmless CNVs (CG-CNVs) are well known since decades. Harmless CG-CNVs can be present as heterochromatic or even as euchromatic variants in clinically healthy persons.

**Results:**

Here I provide a review on what is known today on the still too little studied harmless human CG-CNVs, point out which can be mixed up with clinically relevant pathological CG-CNVs and shortly discuss that the artificial separation of euchromatic submicroscopic CNVs (MG-CNVs) and euchromatic CG-CNVs is no longer timely.

**Conclusion:**

Overall, neither so-called harmless heterochromatic nor so-called harmless euchromatic CG-CNVs are considered enough in evaluation of routine cytogenetic analysis and reporting. This holds especially true when bearing in mind the so-called two-hit model suggesting that combination of per se harmless CNVs may lead to clinical aberrations if they are present together in one patient.

## Background

In 2004 it was a kind of big surprise for geneticists that within the human genome there are hundreds or more regions prone to so-called submicroscopic copy number variations (CNVs) [1–3]. As this kind of CNVs is detectable primarily by molecular genetics, they are abbreviated as MG-CNVs in the following. These MG-CNVs are located in euchromatic regions of, and dispersed over the entire human genome. They are detected by microarray-analyses in each healthy as well as in each (due to other reasons) diseased person [1–4]. Even though still it is not clear if such repeatedly found MG-CNVs have any kind of long term effects on e.g. health, cancer susceptibility, intelligence or life expectance [5–7], at present they are considered as harmless and as not worth to be reported [8].

Before detection of MG-CNVs in 2004 [1–3] it was suggested that no two clinically healthy individuals in human, apart from monozygote twins, are alike due to different gene/allele combinations and point mutations leading to new alleles along the human genome [9]. This is emphasized e.g. by the possibility to perform paternity tests based on single nucleotide polymorphisms [10]. However, studying thousands of patients and normal controls by microarray revealed, that each person distinguishes from another in euchromatic MG-CNVs by the size of up to 1.5 megabasepairs (Mb); also several 0.1 Mb of MG-CNVs are lost or amplified during meiosis from generation to generation [11].

Still, considering what was known and common sense among cytogeneticists on harmless cytogenetically visible CNVs (= CG-CNVs) since decades, the excitement from 2004 is somehow unknowable. Harmless CG-CNVs were first found as heterochromatic variants in the 1960s [12, 13] and later-on in the 1990s even as euchromatic variants (EVs) in clinically healthy persons (for review see [14, 15]). An already early finding of cytogenetics was that on chromosomal level the numbers and kinds of CG-CNVs detected during routine cytogenetics is high; this led to the statement that there are no individuals which are really the same on a chromosomal level, especially concerning the pericentric regions, the acrocentric short arms and - in male - cytoband Yq12 [12, 13, 15]. Thus, the gender-specific interindividual differences in genome size are for sure not only in an average range of only 0.5 Mb as previously suggested [11]; based on variety of heterochromatic CG-CNVs at least 2-4 Mb have to be added.

In the following a review on the overall too little studied, so-called harmless human CG-CNVs is provided, including what is nowadays known on their standard sizes and their anchorage within the human reference genome. As, according to the literature, the major importance of these harmless CG-CNVs is to know the available tools to distinguish them from clinically relevant pathological CG-CNVs, this is also a point covered here. Finally, the question of reporting CG-CNVs and MG-CNVs is discussed in light of the so-called two-hit model, suggesting that combination of at least two per se harmless CNVs may lead to clinical aberrations if they are present together in one individual [11].

### What are harmless human CG-CNVs and where are they localized?

Harmless human CG-CNVs can include heterochromatic and even euchromatic regions. Euchromatic regions are here designated as such which contain genes and are sequence- and alignable. The constitutive heterochromatin, was already earlier defined as “regions that are generally late replicating, rich in repetitive DNA sequences, and genetically inert” [16], and as “that portion of the genome that remains condensed and intensely stained with DNA intercalating dyes throughout the cell cycle. It represents a significant fraction of most eukaryotic genomes and is generally associated with pericentric regions of chromosomes. Contrary to euchromatin, heterochromatic regions consist predominantly of repetitive DNA, including satellite sequences and middle repetitive sequences related to transposable elements and retroviruses. Although not devoid of genes, these regions are typically gene-poor. Establishment of heterochromatin depends on two basic elements: the histonemodification code and the interaction of nonhistone chromosomal proteins” [17].

**Fig. 1:**
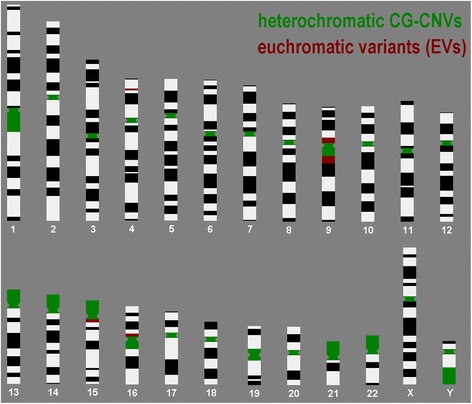
Heterochromatic regions (CG-CNVs) and euchromatic variants (EVs). CG-CNVs and EVs of the human genome are highlighted in a schematically depicted haploid set of human chromosomes

CG-CNVs can be found mostly at specific spots of the human genome as shown in Fig. 1. Heterochromatic CG-CNVs seem to be restricted to pericentric regions of all human chromosomes, all acrocentric short arms and the regions 1q12, 9q12, 16q11.2 and Yq12. Euchromatic CG-CNVs can be divided in such which are repeatedly found and such which are rarely reported. Repeatedly found ones are called “euchromatic variants” (EVs) and are located in 4p16, 8p23.1, 9p12, 9q13-q21.12, 15q11.2 and 16p11.2 (Fig. 1) [14, 15]. Less frequently found euchromatic CG-CNVs, which are not treated here in more detail, include the majority of all pericentric regions and are most often gains of copy numbers due to presence of small supernumerary marker chromosomes (for review see [18]). Finally, CG-CNVs can be present in single cases / families at various regions of the human genome as deletions or duplications (for review see [15]). In the later only occasionally observed cases large euchromatic deletions or duplications (in the range of 5 or more Mb) do not lead to any clinical problems in the corresponding carriers; those are called carriers of unbalanced chromosome abnormalities without phenotypic consequences (UBCA) [14]. It remains to be determined if this is, as in case of EVs due to absence of dosage dependent genes in the affected copy number altered regions of the genome, or due to other reasons, as recently discussed by Crabtree [19, 20] and Mitchell [21]. The text below refers only to harmless euchromatic CG-CNVs which were mentioned before as EVs.

While due to technical reasons heterochromatic CG-CNVs cannot be detected by microarray-technologies, euchromatic CG-CNVs can be found there together with other MG-CNVs. In rare cases MG-CNVs can even have such a size that they become visible on the banding cytogenetic level and are detectable also by molecular cytogenetics [22]. Thus, it has been postulated that MG-CNVs and euchromatic CG-CNVs are biologically the same [15]. The latter may also partly explain the event of rare UBCAs, which, similar to MG-CNVs can be dispersed throughout all the genome (for localization of most frequent MG-CNVs see [23] Fig. 3).

### Standard sizes of harmless human CG-CNVs

Yet there are, due to lack of data, no standard sizes defined for MG-CNVs and for euchromatic CG-CNVs. In other words, it is not known what is to be considered as ‘the normal size’ of those regions [14, 15]. For euchromatic CG-CNVs it can be at least stated that as long as the corresponding regions show a GTG-banding pattern according to the actual international system for human cytogenetic nomenclature [24] they seem to have a size in the normal range. Still, as the resolution of banding cytogenetics is below 5-10 Mb this still leaves space for a wide range of variability. Also, euchromatic CG-CNVs and MG-CNVs, the latter being per definition euchromatic, are anchored in the human reference sequence, i.e. in genome browsers [15], given there as copy number variant, but no standard size is available from there, as well.

For heterochromatic CG-CNVs the story is much more complicated. First of all these regions are not depicted in the human reference sequence. It is argued that this is due to the facts that, (i) nothing is known about the DNA-sequences present there, and (ii) that these regions cannot be sequenced and aligned properly, as they are repetitive. However, both arguments are only partly true. There are studies from 1980, where researchers managed to clone and sequence multiple so-called satellite DNAs derived from the centromeres, the heterochromatic regions on chromosomes 1, 9, 16 and Y and the acrocentric short arms (for review see [15]). Sequences of all centromeric probes used nowadays in molecular cytogenetic diagnostics are known on their base pair level. Besides, many other satellite-DNA-sequences have been reported back in these years, however, later neither studied any more nor included in the genome browsers [15].

Furthermore, even though the international system for human cytogenetic nomenclature [24] was established to achieve a worldwide uniform nomenclature for description of chromosomal alterations, no universal agreement has been included there yet, how to define the standard sizes of (i) centromeres, (ii) heterochromatic regions of chromosomes 1, 9, 16 and Y or (iii) acrocentric short arms. As recently shown it is even worse, and e.g. the sizes of acrocentric short arms vary between different versions of the international system for human cytogenetic nomenclature [15].

**Fig. 2:**
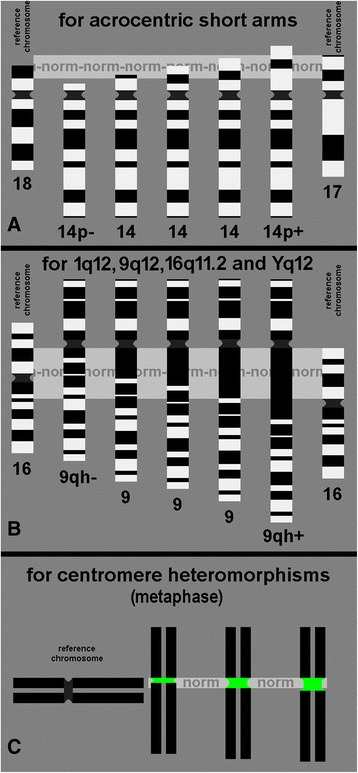
‘Normal sizes’ of heterochromatic regions within the human genome. **a**) Acrocentric short arms are normal size if they have a length between half of 18p and 2/3 of 17p; three different variants all considered as ‘normal’ are depicted for chromosome 14 in the center part of this figure. An example each, for a 14p- and a 14p + are shown left right of these three normal chromosome 14 variants. **b**) For 9q12 three normal sized variants are shown here as before for 14p in figure-part A. Normal sized is for 9p12 if it is between half size of 16p and full 16p. 9qh- and 9qh + are shown as well correspondingly smaller than half of 16p or larger than whole 16p. **c**) For centromeric size the reference size may be the diameter of a chromatide of the same metaphase or stained chromosome. Cen- and cen + heteromorphisms are clearly smaller or larger than a chromatide diameter, respectively

Thus, the following norms were suggested:

- Considered as normal could be for short arm sizes of the acrocentric chromosomes if they are about the same size of the short arm of a chromosome 18 of the same metaphase spread; in other words if it is between half of a chromosome 18p and up to 2/3 of the length of a 17p, the short arm has a normal size. If smaller than half 18p it is a “p-“ variant and if it is larger than 2/3 of a 17p it is a ‘p+’ variant [15] (Fig. 2a);
- for the regions 1q12, 9q12, 16q11.2 and Yq12 normal could be if they have about the same size as the short arm of chromosome 16 of the same metaphase spread. As long as the size is in between half of 16p and complete 16p it is a normal sized region; if smaller than half 16p it is a “qh-“ variant and if larger than 16p it is “qh+” variant [15] (Fig. 2b);
- for centromeric regions visualized by molecular cytogenetics a norm may be that those are about the size of a chromatid (Fig. 2c).

### Frequencies of harmless human CG-CNVs

Incidences of euchromatic CG-CNVs in human population are not available from the literature yet. For MG-CNVs, at least some of them are known to be more frequent than others; still, one has to consider that most available data for MG-CNVs was obtained from Caucasian people, while highest variance is to be expected for Africans [25]. Undoubtedly the MG-CNV present in 15q11.2, also being an EV, is a really common variant seen in many samples in diagnostics; for sure depending on the used reference, it is expresses as gain or loss of copy numbers [26].

As especially heterochromatic CG-CNVs seem to have no direct impact on the phenotype [15], these genomic regions do not underlie the same evolutionary rules as other ones. Thus, these regions may be enlarged or smaller, duplicated, inverted or otherwise rearranged, and these changes may be stabilized within a population [27]. Especially, the short arms of the acrocentric chromosomes being in major parts identical and present in overall 10 copies per cell show nicely this effect. The aforementioned fact that there are practically no single individuals (maybe apart from monozygotic twins), which are really alike on chromosomal level, is mainly due to the variations of acrocentric short arms.

**Fig. 3:**
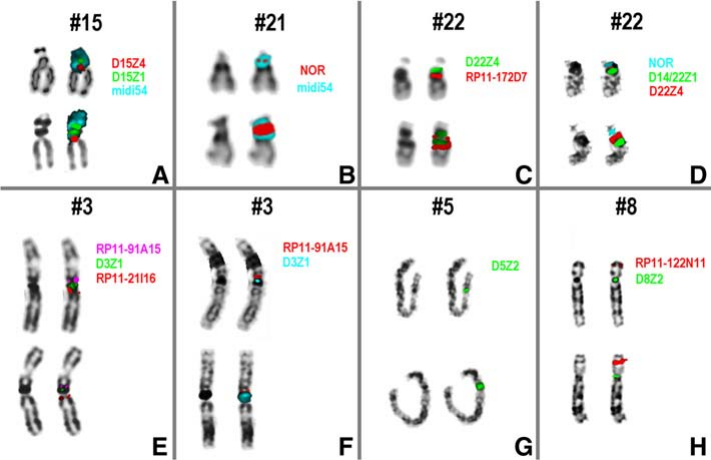
Eight examples of CG-CNVs. Here examples of CG-CNVs are presented as characterized by molecular cytogenetic based hybridization done using probes and protocols as previously reported [15]. All eight studied persons were clinically normal and studied cytogenetically either prenatally, due to infertility or it was a parental analysis due to a clinically affected child. In each part of the figure the studied chromosome pair is indicated at top, the ‘abnormal’ chromosome is shown below the corresponding ‘normal’ homologue and the probes used are indicated right-side of the depicted chromosome. Each chromosome is shown twice: left side just in inverted DAPI-banding and right side fluorescence signals of applied probes on these chromosomes. **a**) A chromosomal enlargement of a short arm of a chromosome 15 was identified as a der(15)(pter- > p11.2::p12- > qter), i.e. an intrachromosomal direct duplication was observed. **b**) The enlarged short arm of a chromosome 21 showed an amplification of NOR-sequences, which can be described according to [15] as der(21)(p12amp). **c**) Similar as in Fig. 3a here a chromosome 22 showed an intrachromosomal direct duplication, however including even parts of cytoband 22q11.21, with a partial karyotype dic(22)(pter- > q11.21::p11.2- > qter). **d**) The result in this case with a strong signal of D22Z4 in 22p11.2 in one and an extremely weak signal of the same probe on the other chromosome 22 was interpreted as a t(22;22)(p11.2;p11.2). **e**) For chromosome 3 DAPI-banding is known to reveal multiple chromosomal heteromorphisms [15]. In this case here chromosome 3 depicted below showed even a conspicuous GTG-banding pattern (not shown). After application of the available pericentromeric probes for chromosome 3 it was obvious that none of the regions covered by those probes was involved in this alteration; still DAPI banding pattern was different and enlarged. Thus the conclusion was that a duplication of satellite I or III DNA reported for that region [15] must be amplified and thus the partial karyotype is: dup(3)(q11.2q11.2). **f**) In this case also GTG-banding already showed an aberrant pattern in the pericentric region of a chromosome 3 (not shown). However, here the probe D3Z1 showed two signal on the derivative chromosome 3. Together with the inverted DAPI-banding pattern an inv(3)(q11.1q11.2) was suggested. **g**) A similar pattern as for the derivative chromosome 3 from Fig. 3f was seen here for a chromosome 5 after applying the alphoid probe D5Z2 (identical to D1Z1 and D19Z3). Still, as D5Z2 is located in 5p11.1 only and an enlargement of DAPI-positive region in 5q was visible a der(5)(pter-> q11.1::p11.1- > p11.1:q11.1- > qter) was reported. **h**) On the chromosome 8 below an altered distal part of the short arm is visible. The probe RP11-122 N11 is specific for the known EV in this region; as is gives a significantly stronger signal on the derivative than on the normal chromosome 8 this prenatal case was considered to carry the known EV without clinical consequences. Later-on a healthy child was born

Even though not based on uniform assessments (see part for standard sizes of CG-CNVs) there is data available on approximate frequencies of harmless heterochromatic CG-CNVs as summarized in Table 1. The most frequently observed chromosomal heteromorphism is an inversion polymorphism of chromosome 9, for which my group previously showed that this includes at least 37 different variants in parts combined with CG-CNVs of this chromosomal region and resolvable by molecular cytogenetics [28]. Given the statement on acrocentric short arms before, it is not surprising that length variations of those are present in ~2.5 % of the general population. Due to technical reasons the acrocentric p- and p + variants could not be further distinguished as cases with inversions, loss of nucleolus organizing regions (NOR), translocations or other rearrangements, even though they exist (see Figs. 3a-d and elsewhere [15]); their frequencies were never studied and/or reported.

**Table 1:** Frequency of CG-CNVs in general human population

Kind of aberration	Frequency [%]
inv(9)(p11q13)	2.86
acrocentric p+	2.38
Yqh+	0.78
16qh+	0.37
9qh+	0.33
1qh+	0.25
acrocentric p-	0.11
inv(2)(p11.2q13)	0.11
Yqh-	0.09

The data was adapted from [15]

### CG-CNVs in diagnostics

From the above mentioned data it may be obvious already, that CG-CNVs may be a more than suited target of future research. Still CG-CNVs play also a role in diagnostics and are matter of discussion concerning the attention which is necessary to be given to them in terms of detailed evaluation and reporting [15, 29].

### Heterochromatic CG-CNVs in metaphase-diagnostics

Examples how heterochromatic CG-CNVs may look like can be found in Fig. 2a-g; more examples can be found elsewhere [15]. Some of them can really make you worried and suggest that there is not only heterochromatin involved but maybe something like balanced or unbalanced translocations behind. Thus molecular cytogenetic studies are necessary to resolve the findings; suited probes may be directed against centromeric regions, 15p11.2 (D15Z1), 22p11.2 (D22Z4), NOR, short arms of all acrocentrics, and Yq12 [15, 30]. Based on such studies one may learn more about how these CG-CNVs may be formed, e.g. by unequal crossing-over events, translocations or amplifications/deletions. Still systematic studies therefore lack [15].

The necessity for thorough characterization, description and reporting of heterochromatic CG-CNVs in diagnostics is highlighted by the following examples:

- Most important is for sure to distinguish between heterochromatic CG-CNVs and semicryptic imbalanced or balanced translocation. An example was previously reported by our group [30] where an enlarged p-arm of a chromosome 13 was indicative for a der(13)t(6;13)(p22.2;p12). Similar cases with semicryptic translocations can also be found elsewhere [15, 31].
- In case of enlargement and/or strange banding pattern of an acrocentric short arm (e.g. Fig. 2a-d) detected in case of a leukemia sample may suggest clinically relevant acquired translocations or oncogene-amplification. In case this was already found earlier and correctly reported as heteromorphism in a previous peripheral blood based chromosomal analyses of the same patient for other reasons, this possibility can be excluded immediately and save important diagnostic time and resources.
- The same holds true for centromeric changes like shown in Figs. 2e-2g, including centromeric enlargement, loss or inversions; misinterpretation in tumor samples can be omitted and additional clarifying studies are not necessary, if the CG-CNV was correctly reported previously.

Still many guidelines recommend not to report such alterations [29, 32], a statement which is only rarely questioned [15].

### Heterochromatic CG-CNVs in interphase-diagnostics

Application of centromeric probes in interphase cells is a major field of molecular cytogenetics [33]. However, without having metaphases in parallel one can never be really sure if the obtained results are interpreted correctly. There are pitfalls reported when applying centromeric probes for chromosomes X, Y and 18 in uncultured amniocytes [34] as well as for a corresponding centromeric probe for chromosome 7 in leukemia [35]. CG-CNVs being relevant here can mimic chromosome loss or gain. Apparent loss can be due to coincidental reduction of alpha-satellite sequences at one tested centromere; apparent gain can be due to by chance amplification of the tested sequences at another, non-homologous chromosome [15, 34, 36]. Thus, in case of interphase-diagnostics without the possibility to study metaphases of the patient, locus-specific probes should always be preferred against heterochromatin-oriented probes.

### Euchromatic CG-CNVs in metaphase-diagnostics

For euchromatic CG-CNVs/EVs two aspects are diagnostically relevant. On the one hand there are such EVs which may be mixed up with adverse copy number changes of the same regions as reported e.g. for the EVs in chromosomes 8 (see Fig. 2h) and 16 [14, 15]. Second, EVs may be a problem in leukemia cases, as EVs may be misinterpreted as translocations, insertions, deletions or oncogene-amplifications.

### CG-CNVs and MG-CNVs in light of the so-called two-hit model

As recently stated, “MG-CNVs are determined as variable copy numbers when compared to a reference genome and may include deletions and duplications of genomic loci. They may encompass as much as 12 % of the human genome. Most of them are considered as benign MG-CNVs and are usually inherited from a parent. When determined as de novo, genomic imbalances are considered more likely pathological. It is also known that each human being carries about thousand MG-CNVs ranging from only a few hundred basepairs to over 1 Mb. The major determinant for the clinical impact of a CNV seems to be, if dosage sensitive genes are present in the corresponding DNA-stretch” [23]. Besides, it was recently found that more than one MG-CNV (larger than 500 kb) can contribute to phenotypic variability associated with genomic disorders and may be the reason for developmental impairment; this phenomenon is called “two-hit”-model [11]. To the best of my knowledge there is no study aligning, or even considering the possibility that euchromatic, and/or (even though being less likely) heterochromatic CG-CNV may contribute to phenotypes or age-related conditions. This is, from my point of view, something to be tested urgently in future! As it is known nowadays that regions of heterochromatic DNA are expressed in early embryogenesis [15, 17] copy number alterations of these regions should have some effect.

## Conclusion

Besides a unique DNA-primary sequence, each human has also an individual combination of CG-CNVs and MG-CNVs; this includes even monozygote twins as differences in MG-CNVs were already found there [37, 38]. If a genetic analyses is performed in a person one has to consider that depending on the chosen test one will always be blind for a part of his genome. If (molecular) cytogenetics is done a genome wide view with low resolution is obtained - including the detection of heterochromatic CG-CNVs. In case of microarray or sequencing based studies a high-resolution of the analyzed genome is the result, but heterochromatic CG-CNVs are missed as well as the information if a copy number change is due to an insertion, translocation or an extra derivative chromosome. Overall, a study in which MG-CNVs and CG-CNVs are evaluated together is still waiting to be done. Combining (molecular) cytogenetics with molecular genetics could help to avoid being blind for possible solutions of yet not understood phenomena in human health.

## Abbreviations

CG-CNVs: Cytogenetically visible CNVs
CNVs: Copy number variations
EVs: Euchromatic variants
Mb: Megabasepairs
MG-CNVs: Submicroscopic CNVs detectable by molecular genetics
UBCA: Unbalanced chromosome abnormalities without phenotypic consequences

## References

[1] Iafrate AJ, Feuk L, Rivera MN, Listewnik ML, Donahoe PK, Qi Y (2004). Detection of large-scale variation in the human genome. Nat Genet..

[2] Sebat J, Lakshmi B, Troge J, Alexander J, Young J, Lundin P (2004). Large-scale copy number polymorphism in the human genome. Science..

[3] Buckley PG, Mantripragada KK (2005). Piotrowski A, Diaz de Ståhl T, Dumanski JP. Copy-number polymorphisms: mining the tip of an iceberg. Trends Genet.

[4] Database of Genomic Variants - A curated catalogue of human genomic structural variation. 2015. http://dgv.tcag.ca/dgv/app/home. Accessed on 11 December 2015.

[5] Grant SF, Hakonarson H (2007). Recent development in pharmacogenomics: from candidate genes to genome-wide association studies. Expert Rev Mol Diagn..

[6] Hong S, Kim Y, Park T (2015). Practical issues in screening and variable selection in genome-wide association analysis. Cancer Inform..

[7] Ku CS, Loy EY, Pawitan Y, Chia KS (2010). The pursuit of genome-wide association studies: where are we now?. J Hum Genet..

[8] South ST, Lee C, Lamb AN, Higgins AW, Kearney HM, Working Group for the American College of Medical Genetics and Genomics Laboratory Quality Assurance Committee (2013). ACMG Standards and Guidelines for constitutional cytogenomic microarray analysis, including postnatal and prenatal applications: revision 2013. Genet Med.

[9] Cinader B (1989). Aging, evolution and individual health span: introduction. Genome..

[10] Sobrino B, Brión M, Carracedo A (2005). SNPs in forensic genetics: a review on SNP typing methodologies. Forensic Sci Int.

[11] Girirajan S, Rosenfeld JA, Cooper GM, Antonacci F, Siswara P, Itsara A (2010). A recurrent 16p12.1 microdeletion supports a two-hit model for severe developmental delay. Nat Genet.

[12] Ferguson-Smith MA, Ferguson-Smith ME, Ellis PM, Dickson M (1962). The sites and relative frequencies of secondary constrictions in human somatic chromosomes. Cytogenetics..

[13] Makino S, Muramoto JI, Tabata S (1966). A survey of a familial transmission of an anomalous autosome in group 13-15. Chromosoma..

[14] Barber JC (2005). Directly transmitted unbalanced chromosome abnormalities and euchromatic variants. J Med Genet..

[15] Liehr T (2014). Benign & Pathological Chromosomal Imbalances.

[16] Jalal SM, Ketterling RP, Wyandt HE, Tonk VS (2004). Euchromatic variants. Atlas of Human Chromosome Heteromorphisms.

[17] Rizzi N, Denegri M, Chiodi I, Corioni M, Valgardsdottir R, Cobianchi F (2004). Transcriptional activation of a constitutive heterochromatic domain of the human genome in response to heat shock. Mol Biol Cell..

[18] Liehr T (2012). Small Supernumerary Marker Chromosomes (sSMC) A Guide for Human Geneticists and Clinicians; With contributions by UNIQUE (The Rare Chromosome Disorder Support Group).

[19] Crabtree GR (2013). Our fragile intellect. Parts I and II. Trends Genet.

[20] Mitchell KJ (2013). Genetic entropy and the human intellect. Trends Genet..

[21] Crabtree G (2013). Our fragile intellect: response to Dr Mitchell. Trends Genet..

[22] Manvelyan M, Cremer FW, Lancé J, Kläs R, Kelbova C, Ramel C (2011). New cytogenetically visible copy number variant in region 8q21.2.. Mol Cytogenet..

[23] Liehr T (2013). Copy number variations - is there a biological difference between submicroscopic and microscopically visible ones?. OA Genetics..

[24] Shaffer LG, McGowan-Jordan J, Schmid M, ISCN (2013). An International System for Human Cytogenetic Nomenclature.

[25] Henn BM, Cavalli-Sforza LL, Feldman MW (2012). The great human expansion. Proc Natl Acad Sci U S A..

[26] Buiting K, Dittrich B, Dworniczak B, Lerer I, Abeliovich D, Cottrell S (1999). A 28-kb deletion spanning D15S63 (PW71) in five families: a rare neutral variant?. Am J Hum Genet..

[27] Genest P (1972). An eleven-generation satellited Y chromosome. Lancet..

[28] Kosyakova N, Grigorian A, Liehr T, Manvelyan M, Simonyan I, Mkrtchyan H (2013). Heteromorphic variants of chromosome 9. Mol Cytogenet..

[29] Brothman AR, Schneider NR, Saikevych I, Cooley LD, Butler MG, Patil S (2006). Cytogenetic heteromorphisms: survey results and reporting practices of giemsa-band regions that we have pondered for years. Arch Pathol Lab Med..

[30] Trifonov V, Seidel J, Starke H, Martina P, Beensen V, Ziegler M (2003). Enlarged chromosome 13 p-arm hiding a cryptic partial trisomy 6p22.2-pter. Prenat Diagn.

[31] Cockwell AE, Jacobs PA, Beal SJ, Crolla JA (2003). A study of cryptic terminal chromosome rearrangements in recurrent miscarriage couples detects unsuspected acrocentric pericentromeric abnormalities. Hum Genet..

[32] Gardner RJM, Sutherland GR, Shaffer LG (2012). Chromosome abnormalities and genetic counseling. Oxford Monographs on Medical Genetcis.

[33] Vorsanova SG, Yurov YB, Iourov IY (2010). Human interphase chromosomes: a review of available molecular cytogenetic technologies. Mol Cytogenet..

[34] Liehr T, Ziegler M (2005). Rapid prenatal diagnostics in the interphase nucleus: procedure and cut-off rates. J Histochem Cytochem..

[35] Duval A, Feneux D, Sutton L, Tchernia G, Léonard C (2000). Spurious monosomy 7 in leukemia due to centromeric heteromorphism. Cancer Genet Cytogenet..

[36] Cockwell AE, Jacobs PA, Crolla JA (2007). Distribution of the D15Z1 copy number polymorphism. Eur J Hum Genet..

[37] Bruder CE, Piotrowski A, Gijsbers AA, Andersson R, Erickson S, Diaz de Ståhl T (2008). Phenotypically concordant and discordant monozygotic twins display different DNA copy-number-variation profiles. Am J Hum Genet.

[38] Mkrtchyan H, Gross M, Hinreiner S, Polytiko A, Manvelyan M, Mrasek K (2010). The human genome puzzle - the role of copy number variation in somatic mosaicism. Curr Genomics..

